# The Dual Role of TNF in Pulmonary Edema

**DOI:** 10.4103/0975-3583.59983

**Published:** 2010

**Authors:** Guang Yang, Jürg Hamacher, Boris Gorshkov, Richard White, Supriya Sridhar, Alexander Verin, Trinad Chakraborty, Rudolf Lucas

**Affiliations:** * *Vascular Biology Center & Department of Pharmacology and Toxicology, Medical College of Georgia, Augusta, GA, 30912, USA*; † *Pulmonary Division, University Hospital, University of Bern, Bern, Switzerland*; ‡ *Department of Pharmacology and Toxicology, Medical College of Georgia, Augusta, GA, USA*; § Institute of Medical Microbiology, Justus-Liebig University of Giessen, Giessen, Germany

**Keywords:** *TNF*, *pulmonary edema*, *cytokine*, *sodium transport*, *hyperpermeability*, *reactive oxygen species*

## Abstract

—Pulmonary edema, a major manifestation of left ventricular heart failure, renal insufficiency, shock, diffuse alveolar damage and lung hypersensitivity states, is a significant medical problem worldwide and can be life-threatening. The proinflammatory cytokine tumor necrosis factor (TNF) has been shown to contribute to the pathogenesis and development of pulmonary edema. However, some recent studies have demonstrated surprisingly that TNF can also promote alveolar fluid reabsorption in vivo and in vitro. This protective effect of the cytokine is mediated by the lectin-like domain of the cytokine, which is spatially distinct from the TNF receptor binding sites. The TIP peptide, a synthetic mimic of the lectin-like domain of TNF, can significantly increase alveolar fluid clearance and improve lung compliance in pulmonary edema models. In this review, we will discuss the dual role of TNF in pulmonary edema.

**Abbreviations:**

—tumor necrosis factor (TNF); acute lung injury (ALI); acute respiratory distress syndrome (ARDS); positive end-expiratory pressure (PEEP);epithelial sodium channel (ENaC);neural precursor cell-expressed developmentally downregulated (gene 4) protein (Nedd4-2);serum and glucocorticoid dependent kinase (Sgk-1);insulin-like growth factor 1 (IGF-1);Protein Kinase C (PKC);reactive oxygen species (ROS);myosin light chain (MLC);pneumolysin (PLY);listeriolysin (LLO);interleukin (IL);bronchoalveolar lavage fluids (BALF);Bacillus Calmette-Guerin (BCG);TNF receptor type 1 (TNFR1); TNF receptor type 2 (TNF-R2);

## PULMONARY EDEMA

Pulmonary edema, defined as an increase in lung water content, occurs when the rate of fluid movement out of the lung’s microvasculature exceeds the capacity of the lymphatics to clear the fluid from the lung’s interstitium^1^. Despite much research pulmonary edema remains one of the more common causes for admission to the hospital and intensive care units^2^.

### 1.1 Mechanisms of Pulmonary Edema Formation

The alveolar–capillary barrier is comprised of capillary endothelium and of alveolar epithelium. Pulmonary alveoli, the primary sites of gas exchange with the blood, are composed of a thin alveolar epithelium (0.1–0.2 µm) that covers 99% of the airspace surface area in the lung and contains thin, squamous type I cells and cuboidal type II cells^3^. Type I cells cover 95% of the alveolar surface and are also the apposition between the alveolar epithelium and the vascular endothelium. This area facilitates efficient gas exchange and forms a tight barrier to fluid and protein movement from the interstitial and vascular spaces, thereby maintaining relatively dry alveoli. Tight junctions connect adjacent epithelial cells near their apical surfaces and maintain apical and basolateral cell polarity. These junctions are critical elements of the permeability barrier required to maintain discrete compartments in the lung^4^. The alveolar type II cell, known for surfactant secretion, is thought to contribute to the vectorial transport of sodium^5^. Active transport of sodium provides a major driving force for fluid removal from the alveolar space. Amiloride-sensitive sodium channels on the apical surface, mainly the epithelial sodium channel ENaC, are involved in fluid transport, with the driving force represented by the Na^+^/K^+^-ATPase on the basolateral surface^3^.

Besides pulmonary epithelia, endothelial barrier function is also a key component for maintenance of the integrity of the vascular boundaries in the lung, particularly since the gas exchange surface area of the alveolar-capillary membrane is large^6^. The endothelial cell lining of the pulmonary vasculature forms a semipermeable barrier between the blood and the interstitium of the lung. Disruption of this barrier can occur during inflammatory disease states, such as pneumonia, acute lung injury (ALI) and the acute respiratory distress syndrome (ARDS). This barrier dysfunction can result in the movement of fluid and macromolecules into the interstitium and pulmonary air spaces, processes which significantly contribute to the high morbidity and mortality of patients afflicted with acute lung injury^7^.

Although many diseases cause pulmonary edema, they do so by means of one or a combination of the following three processes:

- An increased capillary pressure in the lungs;
- An increased permeability or disruption of the alveolarepithelial-endothelial barrier (permeability edema);
- A dysregulated expression or function of crucial ion channels in type II alveolar epithelial cells (type II AEC) implicated in lung liquid clearance, such as the apically expressed epithelial sodium channel ENaC and the basolaterally expressed Na^+^/K^+^-ATPase.

As a consequence, a reduction of lung compliance and an impaired gas exchange may occur, leading to hypoxemia and respiratory acidosis. Patients with pulmonary edema show typical symptoms, including shortness of breath, lung-crackling sounds, pink-stained sputum, cough, anxiety, breathing difficulty, restlessness and wheezing.

### 1.2 Classification of Pulmonary Edema

Pulmonary edema is differentiated into two categories: cardiogenic and non-cardiogenic edema^8^.

#### 1.2.1 Cardiogenic pulmonary edema

Cardiogenic pulmonary edema is defined as pulmonary edema due to increased capillary hydrostatic pressure, secondary to elevated pulmonary venous pressure^9^, it is also called hydrostatic edema or hemodynamic edema^10^. In case of cardiogenic edema, a causal therapy of the underlying disease is often preceded by a symptomatic treatment of the impaired gas exchange, e.g. by means of non-invasive ventilation, paralleled by efficient medical interventions.

#### 1.2.2 Non-cardiogenic pulmonary edema

Non-cardiogenic pulmonary edema occurs due to changes in permeability of the pulmonary capillary or alveolar epithelial membranes, as a result of either a direct or an indirect pathological process and is therefore also known as permeability pulmonary edema^10^. It represents a spectrum of illnesses, ranging from the less severe form of ALI to ARDS. The mainstay of treatment is mechanical ventilation with maximization of ventilation and oxygenation through the judicious use of positive end-expiratory pressure (PEEP). Newer ventilation techniques, such as highfrequency oscillatory ventilation and partial fluid ventilation, are promising but are still in the early stages of clinical testing. Mortality rates unfortunately remain high, despite increased therapy developments in the intensive care unit^8^.

As indicated above, in the clinics, hydrostatic edema is mostly a consequence of heart failure, whereas non-cardiogenic pulmonary edema is mostly a consequence of acute lung inflammation. ARDS is a medical emergency, characterized by the sudden failure of the respiratory system. In 2007, the NHLBI estimated that approximately 190,000 Americans are affected by ARDS annually^11^. Approximately 40% of ARDS cases are fatal, with a mortality rate of even 60% in patients aged 85 years and older^12^. There is accumulating evidence that the capacity of the lung to clear edema liquid is essential for outcome in both cardiogenic and non-cardiogenic pulmonary edema. Patients with ARDS have a dramatically reduced life expectancy when their fluid reabsorption capacity is impaired^13^.

Apart from strategies to optimize ventilation procedures, currently no standard therapy exists for permeability edema. Moreover, viral and bacterial infections can induce a change in the expression or function of epithelial sodium channel (ENaC). Therefore, the search for substances able to reduce the endothelial hyperpermeability and/or restore the sodium uptake in type II AEC is important.

### 1.3 Regulation of ENaC Expression and Activity

The surface expression of ENaC is mainly regulated via the neural precursor cell-expressed developmentally downregulated (gene 4) protein (Nedd4-2), which leads to ubiquitinylation and subsequent degradation of the sodium channel. Specific kinases, such as the cell volume stress-activated serum and glucocorticoid dependent kinase (Sgk-1) and Akt1 (protein kinase B), both part of the insulin and insulin-like growth factor 1 (IGF-1) signaling pathway, were recently proposed to control the surface expression of ENaC, by means of phosphorylating Nedd4-2 and subsequently reducing its binding to ENaC^14^.^15^

Alternatively, Sgk1 has been shown to phosphorylate iNOS in type II alveolar epithelial cells, as such reducing NO production, which inhibits Na+ transport^16^. PI3-kinase can also counteract Protein Kinase C (PKC) activity. PKC is an important negative regulator of ENaC expression. Recently PKC alpha (PKC-α) and zeta isozymes were found to be crucial in ENaC downregulation caused by proteins of SARS-CoV^17^ and of Influenza A virus^18^. Recently, also oxidative stress, which often occurs in the lung under conditions such as infection and inflammation, has been demonstrated to interfere with ENaC expression. Indeed, in lung epithelial cell lines, such as H441 and Calu3 cells, H2O2-mediated oxidative stress was shown to reduce the expression of the alpha subunit of ENaC^19^. In this regard, it is interesting to note that the main etiological agent of community acquired pneumonia, i.e. *Streptococcus pneumoniae*, lacks catalase and therefore secretes H_2_O_2_ as a virulence factor^20^.

### 1.4 Regulation of Endothelial Permeability.

Within endothelial cells, three primary signaling pathways are initiated by the binding of vasoactive factors and leukocyte adhesion: Rho GTPases, reactive oxygen species (ROS), and tyrosine phosphorylation of junctional proteins. These pathways converge to regulate junctional permeability, either by affecting the stability of junctional proteins or by modulating their interactions. The regulation of junctional permeability is mediated by dynamic interactions between the proteins of the adherens junctions, which represent 80% of the tight junctions in endothelial cells and the actin cytoskeleton^21^. Actin/myosindriven contraction generates a contractile force that pulls VEcadherin inward, thus forcing it to dissociate from its adjacent partner, as such producing interendothelial gaps. Another possible mechanism of adherens junctions’ disassembly and interendothelial gap formation involves microtubule disassembly.

A rise in cytosolic Ca^2+^ has been proposed to be the initial pivotal signal preceding endothelial cell contraction, since it can activate key signaling pathways, that mediate cytoskeletal reorganization (through myosin light chain (MLC)-dependent contraction) and disassembly of VE-cadherin at the adherens junctions. Rho (Ras homologous) GTP-binding proteins, which comprise multiple members of the Rho, Rac and Cdc42 subfamilies, are involved in the regulation of a variety of cellular processes^21^. Both RhoA and Rac1 play important roles in the regulation of cytoskeletal remodeling and EC barrier regulation^22^–^27^. RhoA and Rho-associated kinase may directly catalyze MLC phosphorylation or act indirectly via inactivation of MLC phosphatase28,29 to induce cell contraction and endothelial barrier disruption. In turn, endothelial barrier enhancement is associated with Rac 1-mediated formation of F-actin, increased association of focal adhesion proteins, and enlargement of intercellular adherens junctions^7^. Thus, a precise balance between RhoA- and Rac1-mediated signaling is essential for endothelial barrier regulation.

The Ca^2+^-dependent PKC isoform, PKC-α, was suggested to play a critical role in initiating endothelial cell contraction and disassembly of VE-cadherin junctions^21^.^30^ The NADPH oxidases, NOX2 and NOX4, are major sources of ROS in endothelial cells and are implicated in redox-sensitive signaling pathways that influence endothelial cytoskeletal organization and permeability^31^. Apart from inducing RhoA activation^32^, PKC-α activation was also recently shown to upregulate NOX 4 mRNA expression in human endothelial cells.^33^

### 1.5 Role of Bacterial Exotoxins in ALI

Death in severe bacterial pneumonia can occur days after initiation of antibiotic therapy, when tissues are sterile and the pneumonia is clearing and correlates with the presence of bacterial toxins^34^. There is growing evidence that aspects of the immune response greatly contribute to the high mortality rate: while immunosuppressed patients die as a consequence of a poor host response, immunocompetent hosts face overwhelming inflammatory reactions that contribute to tissue injury, shock, and death. In view of its crucial role in bacterial virulence and its profound effects on the immune system of the host, the pore-forming toxin pneumolysin (PLY, from *S. pneumoniae*) and its homologous toxin listeriolysin (LLO, from *Listeria monocytogenes*) can be considered as model toxin for G+ infection-associated acute lung injury and permeability edema.

These toxins bind to cholesterol, followed by oligomerization and membrane pore formation, resulting in a rapid increase in intracellular Ca^2+^ and diacylglycerol levels^35^ and in severe pulmonary hyperpermeability^36^. The interaction of *Streptococcus pneumoniae* with endothelial cells represents a crucial step in its pathogenesis. Intravascular PLY was shown to cause a significant dose-dependent increase in pulmonary vascular resistance and in lung microvascular permeability. By immunohistochemistry, PLY could be detected mainly in endothelial cells of pulmonary arterial vessels, which concomitantly displayed strong vasoconstriction and the toxin moreover increased permeability of HUVEC monolayers^36^. We could recently show that sublytic concentrations of LLO induces cholesterol-dependent actin remodeling by means of interfering with the activity of the small GTP binding proteins RhoA and Rac1 in pulmonary human microvascular endothelial cells^37^.

## CYTOKINES IN PULMONARY EDEMA

### 2.1 Role of Cytokines in Pulmonary Edema

It has been known that inflammation plays an important role in the pathogenesis of pulmonary edema. Once a systemic inflammatory response is triggered, circulating monocytes and alveolar macrophages and neutrophils can secrete cytokines and chemokines, including TNF, interleukin-1β (IL-1β), IL-6, IL-8, IL-12, interferon-γ and IL-8^38^–^40^. Substantial evidence suggests that cytokines are important mediators of the lung injury that follows infection or exposure to microbial products^40^. TNF also plays an important role in the activation of host defense by promoting the production of a wide spectrum of other cytokines and chemokines, such as IL-1, IL-6, IL-8, and granulocyte/macrophage colony stimulating factor in inflammatory processes^41^.^42^ These pro-inflammatory cytokines activate leukocytes and endothelial cells so that these cells increase the expression of surface adhesion molecules. Neutrophils, other leukocytes, and platelets adhere via cognate receptors to the pulmonary endothelium. Activated neutrophils release proteases, leukotrienes, reactive oxygen intermediates, and other inflammatory molecules that amplify the inflammatory response. ROS and proteases can directly damage alveolar–capillary membrane integrity^43^. ROS have also been implicated in ischemia-reperfusion damage following lung transplantation^44^.

Up to date, many studies have shown that TNF can contribute to the pathogenesis and development of pulmonary edema^45^–^53^. Upon checking the cytotoxic effects on the pulmonary endothelium of bronchoalveolar lavage fluids (BALF) from different groups of patients, we found significantly higher TNF levels in the BALF from patients with early-stage ARDS, as compared to control, at risk or late-stage ARDS patients, indicating the implication of this cytokine in barrier dysfunction during the acute phases of the syndrome^54^.

By contrast, new evidence has emerged suggesting that TNF can stimulate lung liquid clearance^55^–^57^ and that the neutralization of this cytokine with neutralizing antibodies can increase the accumulation of edema fluid^58^. Therefore, the role of TNF in the regulation of alveolar liquid clearance and active sodium transport is still controversial, which is why others and our group have tried to unravel its mechanisms of action in edema formation and reabsorption^59^.

### 2.2 Tumor Necrosis Factor (TNF)

TNF was first identified in 1975 as a cytokine with anti-tumor effects *in vitro* and *in vivo*^60^. Extensive research since then has shown that there are at least 18 distinct members of the TNF superfamily exhibiting 15–25% amino acid sequence homology with each other^61^. Among all the members, TNF is the most widely studied pleiotropic cytokine^62^. Although TNF was first identified for its ability to induce rapid hemorrhagic necrosis of cancers^63^, over the years it has become increasingly clear that the cytokine is major component of the inflammatory response and thus its overproduction can play important roles in many diseases of the cardiovascular^64^, respiratory^62^,^65^, endocrinal, metabolic^66^,^67^, nervous 68 and skeletal systems^69^.

#### 2.2.1 The Discovery of TNF

TNF is a potent proinflammatory cytokine produced by many cell types, including monocytes, macrophages, lymphocytes, endothelial cells and fibroblasts^70^. TNF was found in the serum of Bacillus Calmette-Guerin (BCG)-infected mice, and got its name in 1975 by Dr. Old for its ability to mediate endotoxininduced hemorrhagic necrosis^71^. In the early 1980’s the groups of Fiers and Pennica independently cloned TNF cDNA and revealed that it has about 30% homology in its amino acid sequence with lymphotoxin (LT), a lymphokine with similar biological properties^60^,^72^. The similarity between TNF and LT both in sequence and function lead to the renaming of TNF as TNF-α and LT as TNF-β. In the meanwhile, this nomenclature has been changed to TNF and LT-β. Kriegler et al., identified and characterized a rapidly inducible cell surface cytotoxic integral transmembrane form of TNF and named it membrane TNF^73^. In 1997, Black et al., found that this membrane-integrated TNF can be specifically released via proteolytic cleavage by the metalloprotease TNF-alpha-converting enzyme and inactivation of its coding gene in mouse cells caused a marked decrease in soluble TNF production^74^.

#### 2.2.2 The TNF Receptors

Two specific receptors interact with TNF on the cell surface: TNF-R1 (TNF receptor type 1; CD120a; p55/60) and TNF-R2 (TNF receptor type 2; CD120b; p75/80)^75^. TNF-R1 is expressed in most tissues, and can be fully activated by both the membranebound and soluble trimeric forms of TNF, whereas TNF-R2 is found only in cells of the immune system, and respond to the membrane-bound form of the TNF homotrimer^75^. While sharing structural similarities in their extracellular domains, the two TNFRs differ in their intracellular domain, their signal transduction, and consequently their function^76^. The receptorligand interaction causes intracellular signaling without internalization of the complex, which leads to phosphorylation of NF-kB to activate the p50-p65 subunit, which interacts with the DNA chromatin structure to increase transcription of proinflammatory genes, such as IL-8, IL-6 and TNF-α.^77^

#### 2.2.3 The Lectin-like Domain of TNF

Although it is generally assumed that cytokines solely exert their activities upon activating their respective receptors, this does not seem to be completely true in the case of TNF. Indeed, in contrast to Lymphotoxin-alpha, which has a highly homologous 3-D structure as TNF and which is able to bind to both TNF receptors, TNF was shown to exert a lytic activity in purified long slender bloodstream forms of African trypanosomes by means of a lectin-like interaction with oligosaccharide residues on the surface of the parasites^78^. Through investigation, it was found that this lectin-like activity can be attributed to a special domain, named the lectin-like domain of TNF^79^,^80^ which is spatially distinct from its receptor binding sites81. This is further confirmed by the finding that *N, N’*-diacetylchitobiose, a specific binding oligosaccharide of this lectin-like domain, can block this trypanolytic effect whereas it leaves the cytotoxic activity of TNF in cancer cells lines unaffected^81^.

Moreover, a circular seventeen amino acid peptide, named the TIP peptide, (a derivative of the tip region of TNF), mimics the functional structure of this lectin-like domain and has similar lytic effect on African trypanosomes^81^. In recent years, more evidence has been collected demonstrating that this TNF domain is distinct from the two classical receptors, not only regarding its location in the TNF molecule, but also in its functions in different physiopathological processes.

## DELETERIOUS EFFECTS OF TNF IN PULMONARY EDEMA

### 3.1 Effects of TNF on Sodium Uptake Capacity in Type II AEC

Type II alveolar epithelial cells and small airway epithelial cells represent the primary sites for reabsorption of Na^+^ in the lung. Na^+^ ions in the alveolar lining fluid were shown to passively diffuse into fetal distal lung epithelial and alveolar epithelial type II cells through nonselective cationic channels and Na^+^ selective, amiloride-sensitive channels, the most important of which is the epithelial sodium channel ENaC, located in the apical membrane, consisting of at least 4 subunits, i.e. alpha, beta, gamma and delta^82^, with the former one being crucial for its activity^83^. The favorable electrochemical driving force for Na^+^ influx is maintained by the basolaterally expressed, ouabain-sensitive Na^+^/ K^+^-ATPase that transports Na+ into the interstitial space^84^. Epithelial Na^+^ channels represent the rate-limiting step in Na^+^ absorption^85^,^86^. Active Na+ transport across the alveolar epithelium in vivo was proposed to help the reabsorption of fetal fluid after birth and to keep the adult alveolar spaces free of fluid, especially when alveolar permeability to plasma proteins has been increased^87^. Recent data studying mice with reduced ENaC activity also clearly illustrate the impaired lung fluid clearance in these adult mice^88^.

During inflammatory processes in the lungs, proinflammatory substances such as TNF are produced locally. Indeed, it has been demonstrated that there is an elevation of the TNF level in the BALF from patients with ALI/ARDS^48^. Dagenais et al found that TNF decreased the expression of the alpha-, beta-, and gamma-subunits ENaC mRNA to 36, 43, and 16% of the controls after 24 hour treatment and reduced to 50% the amount of alpha-ENaC protein in these cells. There was no impact, however, on Na^+^/K^+^-ATPase mRNA expression. Moreover, in the same study, TNF decreased amiloride-sensitive sodium uptake in a dose-dependent manner^89^. In further investigations, the potential role of TNF on ENaC promoter activity was tested in A549 alveolar epithelial cells; the result showed that TNF decreased luciferase expression by 25% in these cells, indicating that the strong diminution of ENaC mRNA must be related to posttranscriptional events^90^. A recent study suggested that at least in the kidneys, the inhibitory effect of TNF on ENaC expression occurs through a TNF-R1-induced ceramidedependent mechanism^91^. In 2009, Yamagata et al. reported that direct exposure of rat alveolar type II cells to TNF inhibited the mRNA expression of alpha- and gamma-ENaC to 64.0 and 78.0%, but not that of the beta-ENaC; and reduced amiloridesensitive current^59^.

### 3.2 Effects of TNF on Permeability of Epithelialendothelial Barrier

One prominent feature of acute lung injury syndromes is the disruption of the vascular barrier, which can result in permeability pulmonary edema formation and subsequent respiratory dysfunction^92^. The critical importance of the pulmonary vascular barrier function is shown by the balance between competing endothelial cell contractile forces, which generate centripetal tension, and adhesive cell-cell and cellmatrix tethering forces, which regulate cell shape. Both competing forces in this model are intimately linked through the endothelial cytoskeleton, a complex network of actin microfilaments, microtubules, and intermediate filaments, which combine to regulate shape change and transduce signals within and between endothelial cells^7^.

TNF can trigger endothelial cell activation and barrier dysfunction, which are both implicated in the pathogenesis of pulmonary edema^93^. In 1989, Goldblum et al observed that human recombinant TNF could provoke acute pulmonary vascular endothelial injury and increase pulmonary vascular permeability *in vivo* as well as *in vitro*^94^. In another study, TNF is shown to increase the permeability of endothelial cell monolayers to macromolecules and lower molecular weight solutes by a mechanism involving a pertussis toxin-sensitive regulatory G protein^95^. In 1993, Wheatley’s study also showed that TNF could increase the permeability of lung endothelial cell monolayers and that fibronectin could blunt this effect^96^, while Partridge revealed that the TNF-induced increase in endothelial permeability involves the loss of fibronectin and remodeling of the extracellular matrix^97^. It has also been shown that TNF can increase capillary permeability causing transcapillary filtration *in vivo*^98^.

More recent studies have demonstrated that TNF can cause microtubule rearrangement in monolayers of human pulmonary artery endothelial cells^93^.

### 3.3 Other Mechanisms

Besides the direct deleterious effects on Na^+^ channel and barrier dysfunction, it was also reported that TNF can induce pulmonary edema by means of augmenting reactive oxygen species^53^, which has been shown to be able to disrupt pulmonary endothelial barrier99 and to decrease Na+ channel activity^100^.

## PROTECTIVE EFFECTS OF TNF IN PULMONARY EDEMA

### 4.1 Positive Effects of TNF on ENaC Function.

Intriguingly, while many studies have already shown that TNF contributes to the formation of pulmonary edema, other researchers have found that this pro-inflammatory cytokine can actually increase the clearance of alveolar liquid, mainly in infection models.

In 1997, Rezaiguia et al revealed that the instillation of TNF in rats infected with *Pseudomonas aeruginosa* increased alveolar liquid clearance by 43% over 1 h, as compared to control rats. Moreover, when an anti-TNF neutralizing antibody was instilled into the lungs 5 min before the bacteria, alveolar liquid clearance was significantly decreased^56^. The results of Borjesson’s study suggested that intestinal ischemia-reperfusion in a rat model leads to stimulation of alveolar liquid clearance and that this stimulation is mediated, at least in part, by a TNF-dependent mechanism, independent from catecholamine release, because propranolol had no effect and there was no stimulation of cAMP^55^.

In 1997, a triple-mutant murine TNF was generated upon replacement of the crucial residues Thr^104^, Glu^106^, and Glu^109^ with alanines in the lectin-like domain of the TNF trimer. As such, TNF lost its lectin-like affinity, essentially retained its TNF receptor 1-mediated activities, but displayed a 50-fold- reduced TNF receptor 2-mediated bioactivity *in vitro*^101^. We subsequently demonstrated that TNF can increase sodium uptake by an amiloride-sensitive, cAMP-independent mechanism in A549 cells, by means of its lectin-like domain^102^. As shown in Hribar et al., 1999, TNF causes a pH-dependent increase in sodium current in primary lung microvascular endothelial cells and peritoneal macrophages; in a TNF receptor-independent, amiloride-dependent manner, since it also occurs in cells isolated from mice deficient in both TNF receptor types. In this study, the TIP peptide also increased the sodium currents in these cells^103^.

### 4.2 Positive Effects of the Lectin-like Domain of TNF on Permeability of the Epithelial-endothelial Barrier

In isolated endo/exotoxin-treated perfused rabbit lungs, Vadasz et al., recently demonstrated that the TNF-derived TIP peptide significantly lowered vascular permeability, as assessed by capillary filtration coefficient and fluorescein isothiocyanatelabeled albumin flux across the alveolocapillary barrier^104^.

Infections with the G+ bacterium *Listeria monocytogenes* can cause severe lung complications, which can result in permeability edema, characterized by an extensive capillary endothelial hyperpermeability, which requires harsh therapeutic measures and often has a fatal outcome^34^. LLO, the main virulence factor of *Listeria monocytogenes*, induces a dose-dependent hyperpermeability in monolayers of human lung microvascular endothelial cells *in vitro*. In our recent study, The TNF-derived TIP peptide, which mimics the lectinlike domain of the cytokine, was shown to blunt LLO-induced hyperpermeability in vitro, upon inhibiting LLO-induced PKC-α activation, ROS generation and MLC phosphorylation and upon restoring the RhoA/Rac 1 balance. These results indicate that the lectin-like domain of TNF has a potential therapeutic value in protecting from LLO-induced pulmonary endothelial hyperpermeability^37^.

### 4.3 Protective Effects of the Lectin-like Domain of TNF on ROS Generation during Ischemia-Reperfusion Injury

In a recent study, we could demonstrate that the TIP peptide, which mimics the lectin-like domain of TNF, is able to blunt ROS production in pulmonary artery endothelial cells under hypoxia and reoxygenation, and reduces ROS content in the transplanted rat lungs *in vivo*, whereas the inactive mutant TIP peptide didn’t have this effect. Using Ussing chamber experiments of primary type II rat pneumocytes, we concluded that the primary site of action of the peptide is on the apical side of these cells, since only apical, but not basolateral stimulation of the monolayers with the TIP peptide resulted in increased amiloride-sensitive transepithelial currents^44^.

Active Na+ transport across the alveolar epithelium is regulated via not only apically expressed Na^+^ and chloride channels, but also by means of the basolaterally expressed Na^+^/K^+^-ATPase in normal and injured lungs^105^. The study of Vadasz et al, 2008, found that the TIP peptide increased Na^+^/K^+^-ATPase activity 1.6-fold by promoting its exocytosis to the alveolar epithelial cell surface and increased amiloride-sensitive sodium uptake, resulting in a 2.2-fold increase in active Na^+^ transport, and hence improved clearance of excess fluid from the alveolar space^104^. Our Ussing chamber results would rather indicate an indirect role of the Na^+^/K^+^-ATPase in these effects, upon a previous stimulation of ENaC.

## SUMMARY AND FUTURE PERSPECTIVES

Pulmonary edema is still one of the most common medical emergencies, with no standard therapy available for the permeability-induced form of the pathology. As an important cytokine being involved in this pathogenesis, TNF is an example of a “moonlighting protein”, with differential activities mediated by its receptor-binding versus its lectin-like domains, which opens the possibility to design and develop more sophisticated therapeutic regimens to overcome the deleterious fluid accumulation in some major lung pathologies^47^.

In our opinion, the studies mentioned above can generate important advances in our understanding of the complexity of the TNF effects in pulmonary edema. However, in the future, more research is needed in order to reveal the underlying mechanisms of TNF’s protective versus deleterious effects. This research can potentially make the lectin-like domain of TNF an attractive therapeutic option in patients with pulmonary permeability edema.
